# Role of Nitric Oxide in Glioblastoma Therapy: Another Step to Resolve the Terrible Puzzle ?

**Published:** 2014-09-01

**Authors:** R. Altieri, M. Fontanella, A. Agnoletti, P.P. Panciani, G. Spena, E. Crobeddu, G. Pilloni, V. Tardivo, M. Lanotte, F. Zenga, A. Ducati, D. Garbossa

**Affiliations:** 1 Department of Neurosurgery, University of Turin, Italy; 2 Department of Neurosurgery of Brescia, Italy

**Keywords:** Glioma, Glioblastoma, Nitric Oxide, NO synthases, Chemotherapy, Radiothera*y*py

## Abstract

Glioblastoma Multiforme, the most common and aggressive primary brain tumor, remains incurable despite of the advent of modern surgical and medical treatments. This poor prognosis depends by the recurrence after surgery and intrinsic or acquired resistance to chemotherapy and radiotherapy. Nitric oxide is a small molecule that plays a key roles in glioma pathophysiology. Many researches showing that NO is involved in induction of apoptosis, radiosensitization and chemosensitization. Therefore, NO role, if clarified, may improve the knowledge about this unsolved puzzle called GBM.

## I. INTRODUCTION

Glioblastoma Multiforme (GBM), a grade IV astrocytoma, is the most common and aggressive primary brain tumor [1]. Despite the advent of modern surgical and medical treatments, GBM remains an incurable brain disease with a median patient survival time ranging between 12 and 15 months [2–4]. This poor prognosis reflects the prevalence of recurrence after surgery, infiltration into other sites and intrinsic or acquired resistance to chemotherapy and radiotherapy [5, 6].

Currently, treatment plans involve orally administration of temozolomide (TMZ) associated with radiation therapy [7]. TMZ, when used with radiation, has been able to increase median patient survival from 12.1 to 14.6 months in comparison to radiotherapy alone [8].

The chemoresistance is due to many factors that we could divide in extrinsic resistence (like the impossibility of drugs to arrive on the tumor for blood-brain-barrier (BBB)) and intrinsic resistance that is due to biological mechanisms of DNA-repair.

The resistance of tumor cells to the biological effects of alkylating agents like TMZ is due to the DNA repair protein O6-methylguanine-DNA methyltransferase (MGMT) [9, 10]. MGMT is a 22 kDA protein that repairs alkylation at the O6 position of guanine on DNA strands [10]. Unrepaired alkylation results in the induction of apoptosis. Therefore MGMT has an important role against chemotherapeutics agents. In addition to MGMT, another biomolecule that plays an important role in determining chemosensitivity is the protein called p53. It plays a critical role in maintaining the integrity of the genome and determining cellular response, either activating DNA repair mechanisms or triggering apoptosis after exposure to damaging stimuli such as radiation or chemotherapy [11] (table 1).

Ionizing radiation (IR) is widely used as a standard treatment for GBM [12] and an high dose of IR is often fractionated to reduce the side-effects. However, some recent studies have demonstrated that IR paradoxically determine promotion of specific cells in a malignant glioma cell phenotype, determining relapse after treatment [13]. In this context, emerging evidence suggests that a subpopulation of glioma cells is highly tumorigenic and self-renewing, properties reminiscent of normal stem cells [14]. These cells constitute a small percentage of total GBM cells and their gene expression profiles resemble those of normal neural stem cells, therefore they have been called glioma stem-like cells (GSC). Accordingly GSC are thought to be responsible for glioma relapse after treatment and as such are regarded as good potential therapeutic targets [15, 16].

An important modulator of biological therapy response could be Nitric Oxide (NO) and we performed a literature review to investigate and clarified the role of this molecule in GBM therapy.

## II. METHODOLOGY

A literature search using PubMed MEDLINE database has been performed. The search terms “Glioma”, “Glioblastoma”, were combined with “Nitric Oxide”, “NO synthases”. We investigate the role of NO in pathophysiology of glioma and its possible use in therapy.

## III. DISCUSSION

NO is a small, diffusible and short-lived pleiotropic molecule, it plays multiple roles as a messenger within the human body, including the maintenance of the balance between tumor progression and suppression. Under physiological conditions, NO is involved in multiple cellular processes:

- regulation of vasodilation,
- cerebral blood flow,
- vascular permeability may promote tumor cell survival at low concentrations [17–19]
- neurotransmission
- macrophage-mediated immunity [20]

Cells produce NO from arginine using enzymes called nitric oxide synthases (NOS). There are two types of this enzyme: constitutive NOS (cNOS) and inducible NOS (iNOS). cNOS include an endothelial cell form (eNOS) and a neuronal form (nNOS). nNOS is constitutively expressed in brain, eNOS in the endothelium of blood vessels, and iNOS in a variety of cells such as macrophages or glial cells. eNOS and nNOS are calcium-dependent enzymes and generates small amounts of NO phasically. Both constitutively expressed forms and iNOS work as homodimeric enzymes, their activity depend on substrate, L-arginine, and cofactors-coenzymes: NADPH (nicotinamide adenine dinucleotide phosphate) BH4 (tetrahydrobiopterin), FMN (flavine mononucleotide), FAD (flavin adenine dinucleotide), protoporhyrin IX and oxygen. In contrast, iNOS is produced after cells stimulus. Compared to nNOS and eNOS, iNOS enzyme is not regulated by Ca2+ because is not calmodulin dependent even if calmodulin is not covalently bound to iNOS. A large amount of NO is continuously generated by this calcium-independent enzyme. INOS gene expression increases in neuroinflammatory conditions. In vitro studies show that iNOS expression is promoted by different factors such as endotoxin, inflammatory cytochines TNF α, IL 1β A and IFNγ, hypoxic injury, phorbol ester and lipoarabinomannan. More stimulus together contributes to iNOS activation in pathological condition. Inflammatory cytokines promotes iNOS gene transcription: INFγ activates the signal transducer and activator of transcription–1, TNFα and IL-1β induce the activation of another nuclear transcription factor, NF-kb [21]. Many cells types can express iNOS activity, including endothelial cells, astrocytes, neutrophils, and monocytes/macrophages. In vivo, high levels of NO and NOS are found in a great variety of lesions, in peripheral areas of stroke following ischemia/reperfusion and during central inflammatory reactions. All of these enzymes are encoded by a distinct gene [22].

**Figure 1:**
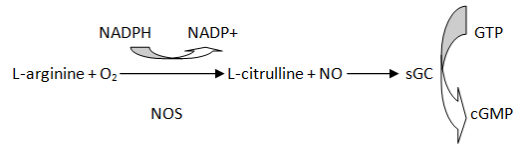


The only known receptor for NO is soluble guanylyl cyclase (sGC). The α1β1 heterodimer is the predominant isoform of sGC that is obligatory for catalytic activity. NO binding at histidine 105 of β1 subunit leads to sGC activity and cGMP production (Fig 1). The NO and 3′,5′-cyclic monophosphate (NO/cGMP) pathway plays a central role in many physiological processes such as induction of vasodilation. On the other hand, the effects of NO can be attributed to the cGMP-independent pathway, which is mediated mainly by reactive oxygen/nitrogen species such as highly reactive peroxynitrite (ONOO) [23]. The role of NO and cGMP signaling in tumor biology has been extensively studied during the past three decades. NO has been shown that affect GBM in great variety of ways [24]. It is almost proved the role of NO in peritumoural and tumoural cortex, and that the increase in NOS activity may play a role in tumour vascularization and progression [25]. Many researches showing that NO treatment results in the induction of apoptosis, radiosensitization and chemosensitization in tumor cells, and in increased permeability of the BBB [26].

Although it is demonstrated that NO exerts anticancer effects on tumor growth, it is also demonstrated that it can also promotes metastasis or inflammation depending on the cellular microenvironment, including the quantity of NO, redox status, cell type and cellular adaptation [27-12]. Thus, a variety of signaling pathways appear to be involved in NO-mediated cellular regulation.

**Figure 2:**
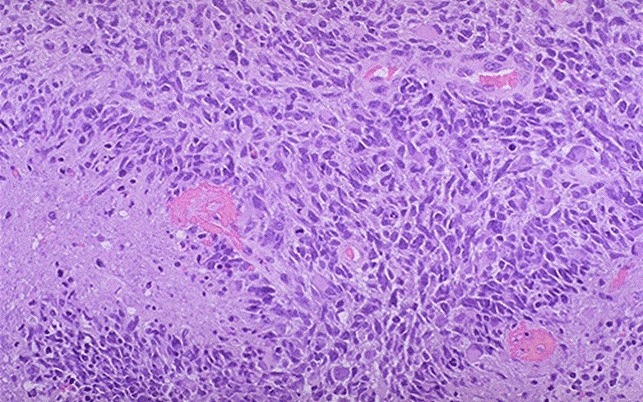
**Glioblastoma (grade IV): vascular proliferation, necrosis, crowded anaplastic cells, marked nuclear atypia, brisk mitotic activity**

Morand & Col. suggest several reasons for ambiguity of NO role in tumor physiopathology: first, although NO participates in physiological signaling (e.g., vasodilation and neurotransmission), NO is also a cytotoxic or apoptotic molecule when produced at high concentrations by iNOS. In addition, the cGMP-dependent (NO/sGC/cGMP pathway) and cGMP-independent (NO oxidative pathway) components may vary among different tissues and cell types (Fig 2). Furthermore, solid tumors contain two compartments: the parenchyma (tumoral cells) and the stroma (supporting tissues including connective tissue, blood vessels, and inflammatory cells that are no malignant) with different NO biology. Thus, the NO/sGC/cGMP signaling molecules in tumors as well as the surrounding tissue must be further characterized before targeting this signaling pathway for tumor therapy [28]. Zhu et Al. found that sGC expression is lower or diminished in human glioma tissues and cell lines and propose that sGC is a novel tumor susceptibility gene in human glioma. Restoring sGC/cGMP signaling genetically or pharmacologically significantly inhibited glioma growth. Orthotopic xenograftment of glioma cells with the α1β1Cys105 sGC stable clone in athymic mice increased the survival time by 4-fold over the control. This increase in survival time exceeds the results recently reported for therapy of glioma with the combination of bevacizumab [Avastin; Genentech (South San Francisco, CA) VEGF-A antibody] and TMZ [29]. Thanks to NO main features (high diffusibility to overpass BBB, short half-life, modulation of perfusion and apoptosis), this molecule could be used in GBM therapy and to get a response modulation of standard GBM therapy. Promising experimental strategies of NO-based therapy for treatment of GBM are focused on the delivery of high NO concentrations into the tumoral cell. Furthermore, these strategies are either focused to increase the drug uptake across the BBB, to sensitize tumor cells to chemotherapeutic drugs like TMN, to reduce radioresistance, and to induce apoptotic cell death selectively in tumor cells. Exogenous NO can be administered using NO-releasing substances (NO donors). Therefore NO donors that release NO for prolonged periods are utilized for therapeutic purposes [30]. Exposure of tumor cells to NO released from NO donors and NO derivates such as peroxynitrite results in accumulation of DNA double strand breaks (DSB), lipid and protein modifications (such as modification of potassium channels by-nitrosylation), and impairment of the mitochondrial energy cycle with resultant breakdown of cellular energy generation [31]. NO released from non-specific NO donors has been shown to induce chemosensitivity in glioma cells [32], but high doses of NO are lesive to healthy tissue.

However, these doses are required for therapeutic efficacy. In literature, there are papers describing in vitro testing on human malignant glioma cells, with specific NO donors targeting only tumoral tissue [34]. Chlorotoxin (CTX), a 36 amino acid protein extracted from the venom of the Death stalker scorpion (Leiurus quinquestraitus), has showed to have an high affinity for matrix metalloproteinase-2 (MMP-2) receptors. It was observed that these receptors are overexpressed in GBM, but not present in the normal brain [34]. CTX can be associated with NO gas in order to form an NO-releasing complex. CTX–NO retains its ability to selectively target glioma cells [35]. In in-vitro assay using human malignant glioma cells, CTX-NO determines a significant reduction in activity levels of MGMT, associated with alteration of p53 activity. These alterations may led to an increase effect of standard chemotherapy whit TMZ, and play a role in decreasing cell invasion [33]. One of the bases of GBM therapy, actually, is fractionated IR therapy. IR induces DNA DSB, which is the most deleterious of DNA lesions, that can lead to cell death if unrepaired [36]. Tumor hypoxia, which occurs mainly as a result mismatch between tumor cell growth and blood supply, is a challenge for successful radiotherapy [37] (table 1). Recent evidence, evaluated on murine tumor model, suggests that IR upregulates NO production and that IR-induced NO has the potential to increase intratumoral circulation. IR also increased eNOS activity and subsequent tissue perfusion in tumors. This led to improvement of tumoral tissue oxygention. Thus, IR-induced NO increased tumor radiosensitivity against further IR exposure [38]. However, other experimental studies in vitro performed on human GBM cells, showed that irradiation of glioma cells also upregulates NO production also from iNOS, which promotes GSC selection. In literature, it has been described that NO is selectively produced in CD133+ glioma cells (GSC), in primary tumor specimens through iNOS, and by this up regulation pattern promotes GSC expansion [39–14]. Taking into consideration other studies, Rae-Kwon Kim, Yongjoon Suhet Al. demonstrate that IR-induced expansion of GSC might reflect dedifferentiation of glioma cells to GSC. These findings could suggest that specific targeting of iNOS associated with IR, might increase the efficacy of radiotherapy for GBM treatment [15].

## IV. CONCLUSION

NO is a small, highly diffusible and short-lived pleiotropic molecule playing multiple and important roles the balance between tumor progression and suppression. In literature we found that NO has a very complex role in the glioma physiopathology. Some evidences show that NO and sGC levels are low in glioma cells and restoring sGC/cGMP signaling, we can inhibit glioma growth. Moreover, NO released from non-specific NO donors has been shown to induce chemosensitivity and radiosensitivity. However, irradiation of glioma cells also up regulates NO production from iNOS and it seems to promotes GSC selection. These findings could suggest that specific delivery of NO on GBM site could decrease TMZ chemoresistance and increase IR efficacy. However, increasing of iNOS could select GST inducing relapse and multiresistance. Accordingly with these findings, we believe that NO should be more investigate because it could play a central role in target therapy of glioblastoma.

## References

[1] Zhang M, Herion TW, Timke C, Han N, Hauser K, Weber KJ, Peschke P, Wirkner U, Lahn M, Huber PE (2011). Trimodal glioblastoma treatment consisting of concurrent radiotherapy, temozolomide, and the novel TGF-beta receptor I kinase inhibitor LY2109761. Neoplasia.

[2] Ohgaki H, Kleihues P (2005). Population-based studies on incidence, survival rates, and genetic alterations in astrocytic and oligodendroglialgliomas. J Neuro pathol Exp Neurol.

[3] Behin A, Hoang-Xuan K, Carpentier AF, Delattre JY (2003). Primary brain tumours in adults. Lancet.

[4] Minniti G, Muni R, Lanzetta G, Marchetti P, Enrici RM (2009). Chemotherapy for glioblastoma: Current treatment and future perspectives for cytotoxic and targeted agents. Anticancer Res.

[5] Altieri R, Agnoletti A, Quattrucci F (2014). Molecular biology of gliomas: present and future challenges. Transl Med UniSa.

[6] Dirks PB (2008). Brain tumor stem cells: bringing order to the chaos of brain cancer. J Clin Oncol.

[7] Robins HI, Chang S, Butowski N, Mehta M (2007). Therapeutic advances for glioblastoma multiforme: Current status and future prospects. Curr Oncol Rep.

[8] Hermisson M, Klumpp A, Wick W, Wischhusen J, Nagel G, Roos W, Kaina B, Weller M (2006). O-6-methylguanine DNA methyltransferase and p53 status predict temozolomide sensitivity in human malignant glioma cells. J Neurochem.

[9] Hansen RJ, Nagasubramanian R, Delaney SM, Samson LD, Dolan ME (2007). Role of O6-methylguanine-DNA methyltransferase in protecting from alkylating agent-induced toxicity and mutations in mice. Carcinogenesis.

[10] Hegi ME, Liu L, Herman JG, Stupp R, Wick W, Weller M, Mehta MP, Gilbert MR (2008). Correlation of O6-methylguanine methyltransferase (MGMT) promoter methylation with clinical outcomes in glioblastoma and clinical strategies to modulate MGMT activity. J Clin Oncol.

[11] Liu Y, Kulesz-Martin M (2001). p53 protein at the hub of cellular DNA damage response pathways through sequence-specific and non-sequence-specific DNA binding. Carcinogenesis.

[12] Bernier J, Hall EJ, Giaccia A (2004). Radiation oncology: a century of achievements. Nat Rev Cancer.

[13] Squatrito M, Brennan CW, Helmy K, Huse JT, Petrini JH, Holland EC (2010). Loss of ATM/ Chk2/ p53 pathway components accelerates tumor development and contributes to radiation resistance in gliomas. Cancer Cell.

[14] Salmaggi A, Boiardi A, Gelati M (2006). Glioblastoma-derived tumorospheres identify a population of tumor stem-like cells with angiogenic potential and enhanced multidrug resistance phenotype. Glia.

[15] Kim Rae-Kwon, Suh Yongjoon (2013). Fractionated radiation-induced nitric oxide promotes expansion of glioma stem-like cells. Cancer Sci..

[16] Sundar SJ, Hsieh JK, Manjila S, Lathia JD, Sloan A (2014). The role of cancer stem cells in glioblastoma. Neurosurg Focus.

[17] Lam-Himlin D, Espey MG, Perry G, Smith MA, Castellani RJ (2006). Malignant glioma progression and nitric oxide. Neurochem. Int..

[18] Ridnour LA, Thomas DD, Donzelli S, Espey MG, Roberts DD, Wink DA, Isenberg JS (2006). The biphasic nature of nitric oxide responses in tumor biology. Antioxid. Redox Signal.

[19] Wink DA, Mitchell JB (2003). Nitric oxide and cancer: an introduction, Free Radic. Biol Med.

[20] Fukumura D, Kashiwagi S, Jain RK (2006). The role of nitric oxide in tumour progression. Nat Rev Cancer.

[21] Tardivo V, Crobeddu E, Pilloni G, Fontanella M, Spena G, Panciani PP, Berjano P, Ajello M, Bozzaro M, Agnoletti A, Altieri R, Fiumefreddo A, Zenga F, Ducati A, Garbossa D (2014). Say “no” to spinal cord injury: is nitric oxide an option for therapeutic strategies?. Int J Neurosci.

[22] Bakshi A, Nag TC, Wadhwa S, Mahapatra AK, Sarkar C (1998). The expression of nitric oxide synthases in human brain tumours and peritumoral areas. J Neurol Sci.

[23] Bian K, Murad F (2003). Nitric oxide (NO)–biogeneration, regulation, and relevance to human diseases. Front Biosci.

[24] Fukumura D, Kashiwagi S, Jain RK (2006). The role of nitric oxide in tumour progression. Nat Rev Cancer.

[25] Garbossa D, Fontanella M (2001). Nitric oxide synthase and cytochrome c oxidase changes in the tumoural and peritumoural cerebral cortex. Acta Neurochir (Wien).

[26] Kogias E, Osterberg N, Baumer B, Psarras N, Koentges C, Papazoglou A, Saavedra JE, Keefer LK, Weyerbrock A (2012). Growth-inhibitory and chemosensitizing effects of the glutathione-S-transferase-p-activated nitric oxide donor PABA/NO in malignant gliomas. Int J Cancer.

[27] Hussain SP, Trivers GE, Hofseth LJ (2004). Nitric oxide, a mediator of inflammation, suppresses tumorigenesis. Cancer Res.

[28] Bian K, Murad F (2014). What is next in nitric oxide research? From cardiovascular system to cancer biology. Nitric Oxide.

[29] Zhu H, Li JT, Zheng F, Martin E, Kots AY, Krumenacker JS, Choi BK, McCutcheon IE, Weisbrodt N, Bögler O, Murad F, Bian K (2011). Restoring soluble guanylyl cyclase expression and function blocks the aggressive course of glioma. Mol Pharmacol.

[30] Muscara MN, Wallace JLV (1999). Therapeutic potential of nitric oxide donors and inhibitors. Am J Physiol Gastrointest Liver Physiol.

[31] Leon L, Jeannin JF, Bettaieb A (2008). Post-translational modifications induced by nitric oxide (NO): implication in cancer cells apoptosis. Nitric Oxide.

[32] Weyerbrock A, Baumer B, Papazoglou A (2009). Growth inhibition and chemosensitization of exogenous nitric oxide released from NONOatesin glioma cells in vitro. J Neurosurg.

[33] Safdar S, Payne CA (2013). Targeted Nitric Oxide Delivery Preferentially Induces Glioma Cell Chemosensitivity via Altered p53 and O6-Methylguanine-DNA Methyl transferase Activity. Biotechnol Bioeng.

[34] Deshane J, Garner CC, Sontheimer H (2003). Chlorotoxin inhibits glioma cell invasion via matrix metalloproteinase-2. J Biol Chem.

[35] Safdar S, Taite LJ (2012). Targeted diazeniumdiolates: Localized nitric oxide release from glioma-specific peptides and proteins. Int J Pharm.

[36] Jackson SP, Bartek J (2009). The DNA-damage response in human biology and disease. Nature.

[37] Hall EJ, Giaccia AJ (2011). Radiobiology for the Radiologist.

[38] Naganea M, Yasui H (2013). Radiation-induced nitric oxide mitigates tumor hypoxia and radioresistance in a murine SCCVII tumor model. Biochemical and Biophysical Research Communications.

[39] Eyler CE, Wu Q, Yan K (2011). Glioma stem cell proliferation and tumor growth are promoted by nitric oxide synthase-2. Cell.

